# Editorial: Innovative approaches in remote sensing for precise crop yield estimation: advancements, applications, and future directions

**DOI:** 10.3389/fpls.2026.1816342

**Published:** 2026-03-10

**Authors:** Aichen Wang, Imran Ali Lakhiar, Altaf Ali Siyal

**Affiliations:** 1 School of Agricultural Engineering, Jiangsu University, Zhenjiang, Jiangsu, China; 2 Faculty of Agricultural Engineering and Technology, Sindh Agriculture University, Tandojam, Sindh, Pakistan

**Keywords:** AI for agriculture, crop monitoring, crop yield estimation, drones, image processing, remote sensing, UAVs

Agriculture currently faces the dual pressures of ensuring global food security and adapting to rapid climate change. To cope with these challenges, researchers have introduced several modern mechanization technologies, including advanced farm machinery, autonomous navigation systems, artificial intelligence, sensing technologies, and communication tools, to enhance productivity and sustainability (Syed et al., 2025a). These technologies enable data-driven decision-making by allowing continuous, large-scale acquisition and analysis of crop and environmental information. Consequently, accurately predicting crop yields and monitoring plant health in real time have become critical prerequisites for precision agricultural management (Syed et al., 2025). Traditional measurement methods—often labor-intensive, destructive, and spatially limited—are increasingly unable to meet the demands of modern large-scale farming. In this context, the integration of Remote Sensing (RS) with Artificial Intelligence (AI), particularly Deep Learning (DL), has triggered a technological paradigm shift.

This Research Topic focuses on advancing understanding latest breakthroughs in this interdisciplinary field, illustrating a clear trajectory from simple observation to intelligent, robust, and physiological decision-making. A total of 13 manuscripts were submitted, of which 10 articles were published, including 9 original research articles and 1 review article. These studies involve AI-Powered detection of pumpkin leaf diseases using dualfusion model, multimodal cross-attention network for strawberry seedlings, YOLOv8-FDA lightweight detection model for Wheat Ear detection, dynamic coding network for robust fruit detection, refining the estimation of the fraction of photosynthetically active radiation (FPAR) within the CASA model, a UAV-based multitier feature selection improves nitrogen content in cotton, DUNet, a high-performance image dehazing model, a light use efficiency model with a random forest approach to predict the mean crop biomass and proposed the TA-YOLO modeling framework, aimed at improving the efficiency and accuracy of small tomato fruit detection. In addition, a significant portion of this Research Topic addresses the challenge of detecting small, dense, or occluded targets in complex agricultural environments. The evolution from standard computer vision to specialized, lightweight architectures is evident. For cereal crops, Lin et al. introduced YOLOv8-FDA, a lightweight model designed for wheat ear counting using drone imagery. By integrating the RFAConv and DySample modules, they successfully addressed feature confusion in high-density planting, achieving high precision with a model size under 3MB, making it ideal for edge deployment. In the horticultural sector, Zhao et al. proposed Ta-YOLO to tackle the “target blocked” challenge in greenhouse tomato detection, utilizing a Multi-dimensional Attention Structure (CSAM) to significantly improve the recognition of fruits hidden by dense foliage. Similarly, aiming at robotic harvesting in outdoor orchards, Lu et al. developed the Dynamic Coding Network (DCNet), which utilizes an Iterative Feature Attention mechanism to robustly detect fruits like persimmons and grapes even under low-visibility conditions caused by shadows or fog. Complementing these detection advances, Huang et al. provided a comprehensive review of visual perception technology for harvesting robots, summarizing the state-of-the-art in sensors and algorithms that enable robots to operate in such unstructured environments.

Beyond simple object counting, the Research Topic highlights the power of fusing different data sources to estimate physiological traits and crop health. Cheng et al. Developed MM-CAPNet, a multimodal cross-attention network for the early detection of overgrowth in strawberry seedlings. By fusing visual images with historical environmental time-series data, the model effectively links visual phenotypes with their environmental drivers, offering a tool for precise nursery management. In the realm of phytoprotection, Bhuria et al. presented a hybrid deep learning framework, DualFusion-CBAM-Stochastic, for detecting pumpkin leaf diseases. By fusing DenseNet121 and EfficientNetB3 backbones with attention mechanisms, they achieved superior accuracy in classifying diseases, demonstrating the potential of AI in early disease intervention. Field crop physiology was also a key focus, as Li et al. addressed nitrogen management in arid-region cotton. Using UAV multispectral sensing and a Boruta-SHAP feature selection method, their Bayesian-optimized Random Forest model successfully estimated leaf nitrogen concentration despite the interference of plastic mulch.

Finally, the scope of this topic extends to environmental robustness and landscape-scale analysis, ensuring that these technologies work in the “messy” reality of farming. To address image quality issues, Zhao et al. Introduced DUNet, a novel dehazing model grounded in atmospheric-scattering physics. This model effectively restores color and detail in hazy UAV images, significantly extending the operational window for agricultural drones in humid climates. At a broader scale, Li et al. improved the estimation of Net Primary Productivity (NPP) for corn and rice by integrating high-resolution Sentinel-2 imagery with a Convolutional Neural Network into the CASA model, significantly reducing estimation errors compared to traditional regression methods. Furthermore, Dhillon et al. analyzed how landscape structure, climate variability, and soil quality shape crop biomass in Bavaria. Their findings that landscape diversity (e.g., hedgerows) positively influences yield stability highlight the critical importance of integrating “ecological intensification” with technological precision.

Together, these ten contributions illustrate the maturation of agricultural remote sensing, moving towards models that are not only more accurate but also lighter, more interpretable, and more resilient to environmental noise. By combining satellite and UAV data with advanced computational models, these innovative approaches are paving the way for a more resilient and productive global food system.

Future research will increasingly focus on improving the precision of crop yield estimation models through multi-dimensional analyses. As agricultural environments grow more complex, integrating AI-powered models with multi-sensor fusion technologies will be essential. Innovations such as lightweight neural networks and multimodal cross-attention frameworks will enable the detection of small, occluded, and densely packed targets with greater accuracy, thereby refining crop-specific metrics such as photosynthetically active radiation (FPAR) and nitrogen content. This, in turn, will enhance crop health monitoring and yield predictions.

Additionally, UAV-based remote sensing, combined with multitier feature selection, will improve nitrogen content analysis in crops such as cotton, while image dehazing models and light-use efficiency frameworks will bolster biomass estimation. Emerging technologies such as the Ta-YOLO framework will further optimize small fruit detection in dense canopies, advancing overall crop detection accuracy.

A key challenge lies in adapting these models to handle real-world complexities, such as variable environmental conditions. Future work will focus on improving the robustness of these models through dynamic coding networks and performance optimization, ensuring they can operate in heterogeneous agricultural environments. Interdisciplinary collaboration between agriculture, AI, and remote sensing experts will accelerate the development and deployment of these approaches, paving the way for more efficient crop yield estimation systems that are critical for ensuring food security and sustainable agricultural practices.
